# Mention effect in information diffusion on a micro-blogging network

**DOI:** 10.1371/journal.pone.0194192

**Published:** 2018-03-20

**Authors:** Peng Bao, Hua-Wei Shen, Junming Huang, Haiqiang Chen

**Affiliations:** 1 School of Software Engineering, Beijing Jiaotong University, Beijing, China; 2 CAS Key Laboratory of Network Data Science and Technology, Institute of Computing Technology, Chinese Academy of Sciences, Beijing, China; 3 CompleX Lab, Web Sciences Center and Big Data Research Center, University of Electronic Science and Technology of China, Chengdu, China; 4 Center for Complex Network Research, Northeastern University, Boston, MA, United States of America; 5 China Information Technology Security Evaluation Center, Beijing, China; Nagoya University, JAPAN

## Abstract

Micro-blogging systems have become one of the most important ways for information sharing. Network structure and users’ interactions such as forwarding behaviors have aroused considerable research attention, while *mention*, as a key feature in micro-blogging platforms which can improve the visibility of a message and direct it to a particular user beyond the underlying social structure, is seldom studied in previous works. In this paper, we empirically study the mention effect in information diffusion, using the dataset from a population-scale social media website. We find that users with high number of followers would receive much more mentions than others. We further investigate the effect of mention in information diffusion by examining the response probability with respect to the number of mentions in a message and observe a saturation at around 5 mentions. Furthermore, we find that the response probability is the highest when a reciprocal followship exists between users, and one is more likely to receive a target user’s response if they have similar social status. To illustrate these findings, we propose the response prediction task and formulate it as a binary classification problem. Extensive evaluation demonstrates the effectiveness of discovered factors. Our results have consequences for the understanding of human dynamics on the social network, and potential implications for viral marketing and public opinion monitoring.

## Introduction

Nowadays, the subject of information diffusion is central to an information era from knowledge database to online media [1–4]. Information diffusion is a fundamental process in social network, capturing behaviors that cascade from node to node like an epidemic or chain reaction. Recent studies devote to investigating the diffusion process of different type of information, such as text [5], image [6], video [7], etc. On the micro-blogging platforms such as Twitter and Sina Weibo, users can post any topic of messages no longer than 140 characters and follow any other users to receive their messages. Moreover, with various sharing features on the platform, every user owns the power to effectively spread information beyond the underlying followship structure [8, 9]. In recent years, researchers have paid great effort on user interest modeling [10–12], influential users identification [13–15] and recommendations [16–19]. Understanding the mechanisms of information diffusion is especially critical in a wide range of areas, such as human dynamics [20–22], popularity prediction [5, 23–26], and viral marketing [27, 28].

The characteristics of network structure and user relationship of micro-blogs have aroused considerable research interests in the past few years [29, 30]. Recently, researchers have paid extensive attention on characterizing information cascades [31, 32], discovering structural and temporal patterns [33–35], and further predicting individual behaviors and popularity dynamics [36–39]. However, previous works mainly focused on the re-tweeting or forwarding behaviors, assuming that information diffusion relies on the underlying followship network among users. One can be exposed to a message only when he/she has already followed the publisher or spreader of the message. Therefore, the scale of the diffusion would be limited due to the visibility restriction [40, 41].

However, as a key feature in micro-blogging platforms, *mention* can improve the visibility of a message and direct it to a particular user beyond the underlying social structure. A user uses the “@username” to mention another user in the body of a message, so that the be-mentioned user will see the message in his/her personal mention tab. One’s followers would also easily miss a message in time if there’s no notification [13]. Therefore, with the proper usage of mention, an ordinary user has the potential to break through the visibility barrier and spread his/her messages broadly. In recent years, the essential question of whom-to-mention in a message has been studied extensively. Most previous works formulate the problem as a ranking based recommendation task [42–46], while some researchers take it as a link prediction problem [47] or an unbalance assignment problem [48]. Besides, different aspects of factors have been investigated, such as content [44, 46, 49], social influence [46, 50], spatiotemporal information [44, 48, 51], and the interests of users [50, 52]. However, the underlying microscopic factors governing the effectiveness of mention still need to be explored. Therefore, it is still an open problem and of great interest to present an in-depth study of mention effect in information diffusion on social networks.

In this paper, in order to investigate the mention effect in information diffusion on micro-blogging networks, a comprehensive empirical study is conducted on the most popular micro-blogging website in China, namely Sina Weibo. Note that here the unit of information refers to a message on the micro-blogging network, and we use the forwarding behaviors among users as the proxy for information diffusion, which is also widely adopted in previous studies [23, 53]. We start with the statistical characteristics of mention in information diffusion represented as diffusion network. We find that users with high number of followers would receive much more mentions than those small-degree users, and meanwhile it brings the problem of mention overload. We further investigate the effect of mention in information diffusion by examining the response probability from the perspective of mention count and network structure respectively. We observe a saturation at around 5 mentions in a message which means that with each additional mention, a message is more and more likely to receive response from the mentioned users, up to a point. When a message contains more than 5 mentioned users, the response probability increase marginally. Furthermore, we examine the response probability with respect to the network structure between users. We find that the response probability is the highest when a reciprocal followship exists between users. In addition, one is more likely to receive a target user’s response if they have similar social status. To illustrate these findings, we propose the response prediction task and formulate it as a binary classification problem. Extensive evaluation demonstrates the effectiveness of discovered factors.

## Results

### Diffusion network

**Fig 1:**
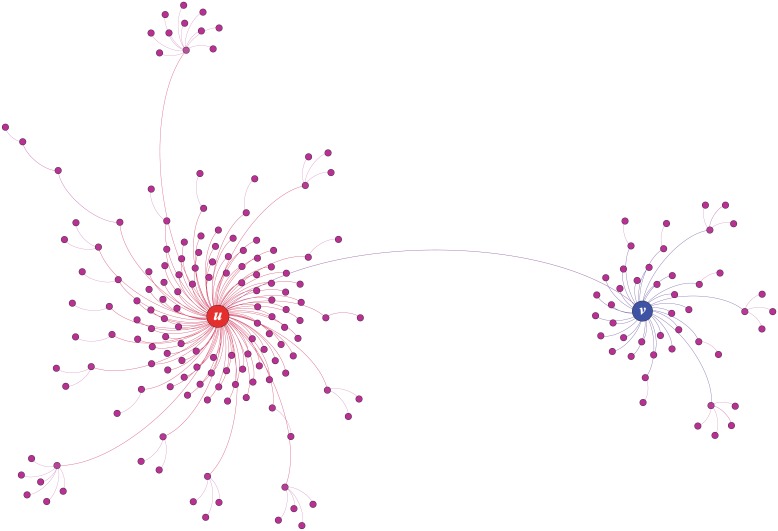
Diffusion network of a message containing mentions. This example is derived from a real cascade. The node *u* initiated the cascade of the message and mentioned the node *v*. We observed that the be-mentioned node *v* forwarded the message and further triggered a new spread of the message.

To begin our analysis, the cascade of message is represented as a diffusion network which characterizes the relationship among users who involve in the diffusion process. In this paper, we construct the diffusion network as a directed network where each node represents an involved user in the diffusion process and each link denotes an observed forwarding behavior between two users, as done in [53]. In addition, we call the node which corresponds to the source user of message the root node of diffusion network. Fig 1 gives an example of diffusion network of a message containing mentions, which is derived from a real cascade. The root node *u* initiated the cascade of the message with a mentioned user which is marked by the blue node *v*. Soon after being mentioned, node *v* forwarded the message and further triggered a new spread of the message. In this paper, we define the be-mentioned user’s forwarding behavior towards the message as ***response***.

**Fig 2:**
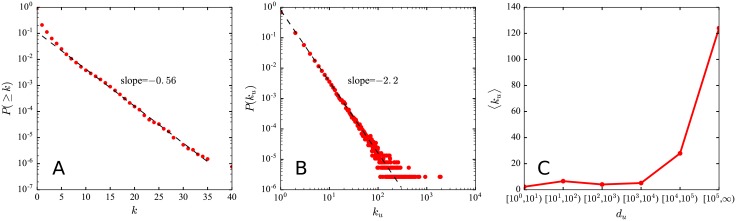
Statistical characteristics of mention. **(A)** Cumulative distribution *P*(≥ *k*) where *k* denotes the number of ‘@’ in a message. The cumulative distribution of *k* is exponential with an exponent 0.56. We can also observe that more than 20% of messages are posted with at least one mentioned user. **(B)** Distribution *P*(*k*_*u*_) where *k*_*u*_ denotes the number of ‘@’ user *u* received. It indicates a power law interdependence with an exponent 2.2. **(C)** Average number of received ‘@’ 〈*k*_*u*_〉 versus the number of followers *d*_*u*_ for each user *u*. We classify users into six categories according to *d*_*u*_. We find that users with large number of followers receive much more ‘@’ than small-degree users.

We first investigate the statistics of the number of ‘@’ in a message. As depicted in Fig 2A, more than 20% of messages are posted with at least one mentioned user. Due to the strict length restriction of a message, only a small number of users can be mentioned in a message, which follows an exponential distribution with an exponent 0.56. We further examine the distribution of the number of ‘@’ a user would receive. From Fig 2B, we can observe a power law distribution with an exponent 2.2, indicating that mention is allocated in a rather asymmetric way, with a majority of users getting a few mentions, whereas a few receive a disproportionate number of mentions. Users with high number of followers on the explicit followship network are usually called “opinion leaders” [54] and are indispensable to the popularity of a message. Hence they would receive much more mentions than those small-degree users, as shown in Fig 2C. However, a user being mentioned too many times will suffer from the severe mention overload problems. Tons of mention notifications will interrupt user’s daily use of micro-blogs and decrease user’s interest in forwarding.

### Effect of mention count

Given the statistical characteristics of mention in information diffusion, here we ask: How about the effect of mention? Are there any factors that would affect mention effect? Can we predict the response of those be-mentioned users? To address these questions, we first quantify the effect of mention in terms of ***response probability***. Specifically, for a given message with *k* mentioned users, we define response probability *p*(*k*) as the probability that at least one of the *k* mentioned users will forward the message. Here we just consider the forwarding behavior as a sign of direct response. We denote *M*(*k*) the number of messages with *k* be-mentioned user, and *R*(*k*) the number of messages that receive response by at least one of the *k* mentioned users. We then conclude that $p\left(k\right)=\frac{R(k)}{M(k)}$ is the corresponding response probability.

**Fig 3:**
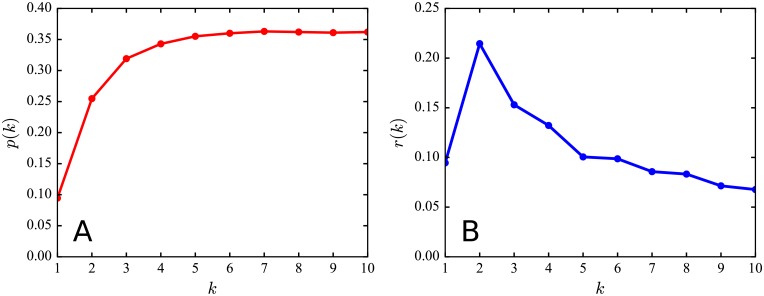
Effect of mention count. **(A)** Response probability *p*(*k*) versus mention count *k* in a message. We find that response probability increases with more and more mentions in a message, up to a saturation point around *k* = 5. **(B)** Response ratio *r*(*k*) versus mention count *k* in a message. We can observe a peak in response ratio at 2 mentions in a message and then a slow drop.

One would expect that a message is more likely to receive response if it contains more mentions. On the other hand, one would also think that there is a saturation point. With the above definition, we empirically study the response probability *p*(*k*) using all messages forwarded by more than 10 users. Taking the activity pattern of users on the platform into consideration, we thus only consider messages with the post time between 10am and 10pm per day, as done in [53]. Fig 3A shows *p*(*k*) with respect to the number of mentions *k* in a message. We observe a saturation at around 5 mentions. This means that with each additional mention, a message is more and more likely to receive response from the mentioned users, up to a point. When a message contains more than 5 mentioned users, the response probability increase marginally.

Furthermore, we examine the percentage of response users among all be-mentioned users in a message. For a message with *k* mentions, and *n*_*r*_ is the number of be-mentioned users who give response to the message. We define the *response ratio* of the message as *n*_*r*_/*k*. We use *r*(*k*) to represent the average response ratio over all messages with *k* mentions. As shown in Fig 3B, we can observe a peak in response ratio at 2 mentions in a message and then a slow drop. This implies that if a message contains more than two mentions, the be-mentioned users are less likely to give response to it, possibly because a message mentioning a lot of users is likely to be supposed as a spam, which will decrease others’ interest in forwarding it. We further investigate the response ratio with respect to different kinds of be-mentioned users, which reveals that users with large number of followers are less likely to respond to a mention probably due to the overload of mentions (S1 Fig).

### Effect of network structure

When going beyond the mention effect on the mention count in a message, we continue to wrestle with response probability with respect to the network structure between users.

**Fig 4:**
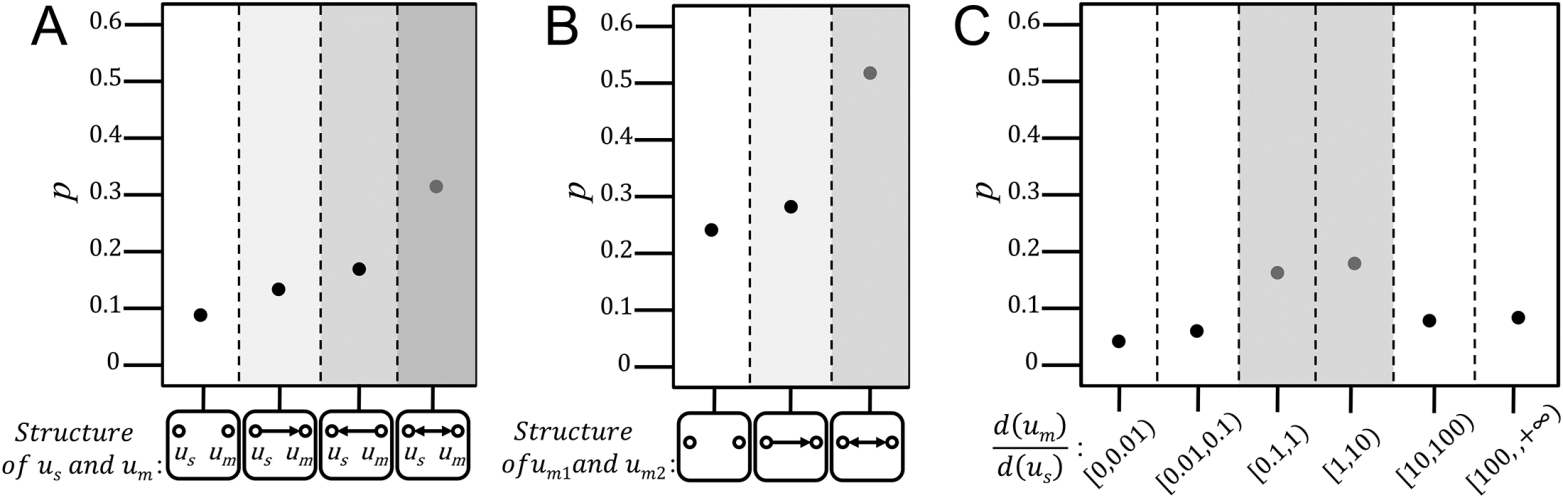
Effect on network structure. **(A)** Response probability *p* versus structure of source user *u*_*s*_ and be-mentioned user *u*_*m*_. **(B)** Response probability *p* versus structure of two be-mentioned users *u*_*m*1_ and *u*_*m*2_. From (a)(b), we can both observe that the response probability is the highest when a reciprocal followship exists between users. **(C)** Response probability *p* versus degree ratio $\frac{d(u_{m})}{d(u_{s})}$, where *d*(*u*_*m*_) represents the number of followers of be-mentioned user *u*_*m*_ and *d*(*u*_*s*_) is the number of followers of source user *u*_*s*_. We find that the response probability of the degree ratio between [0.1, 10) is higher than the others.

We start with the topological structure between source user *u*_*s*_ and be-mentioned user *u*_*m*_. According to their followship on the social network, we have four types of structures: (a) no followship between *u*_*s*_ and *u*_*m*_, (b) *u*_*s*_ follows *u*_*m*_, (c) *u*_*m*_ follows *u*_*s*_, and (d) reciprocal followship between *u*_*s*_ and *u*_*m*_. Note that followship offers a proxy for tie strength while the reciprocal followship represents a strong tie of friendship between users [55]. Fig 4A demonstrates the response probability with respect to the four types of structures. We can observe that the response probability of the reciprocal followship is significantly higher than the others, demonstrating that the stronger tie strength between two users, the more likely for one user to receive response via mentioning the other in a message.

Moreover, we investigate the interdependence between the response probability and the network structure among be-mentioned users. Among all the cases where a message contains multiple mentions, 2–mention case is the most frequent one and the study of response probability for 2–mention case can be easily extended to other cases of multiple mentions. Therefore, in this paper, we only focus on the 2–mention case. For convenience, we denote the two be-mentioned users as *u*_*m*1_ and *u*_*m*2_. Then, according to the followship between *u*_*m*1_ and *u*_*m*2_, we have three types of structures: (a) no followship between *u*_*m*1_ and *u*_*m*2_, (b) *u*_*m*1_ follows *u*_*m*2_ or *u*_*m*2_ follows *u*_*m*1_, and (c) reciprocal followship between *u*_*m*1_ and *u*_*m*2_. Intuitively, the more number of followships among users, the more overlapped their interest will be, and therefore the more probable they will interact with each other. From Fig 4B, we find that the response probability of the reciprocal followship structure is the highest. This finding implies that mentioning connected users in a message would probably motivate them to participate in the discussion and therefore respond to it.

Finally, we also examine whether the status difference between two users will affect the mention effect. Here we adopt the number of followers as a measure of a user’s status. We define the *degree ratio* as $\frac{d(u_{m})}{d(u_{s})}$, where *d*(*u*_*m*_) represents the number of followers of the be-mentioned user *u*_*m*_ and *d*(*u*_*s*_) is the number of followers of the source user *u*_*s*_. As shown in Fig 4C, the response probability of the degree ratio between [0.1, 10) which could be viewed as similar status between *u*_*m*_ and *u*_*s*_, is higher than the others. This means that one is more likely to receive a target user’s response if they have similar social status. One possible explanation for these findings is that people are living in status groups and they are only supposed to engage with people of like status [56].

### Response prediction

To illustrate the empirical findings, we turn to the question: Can we predict the response of those be-mentioned users? We call this problem as “response prediction” (*RP*). Formally, given a message *d*, the source user *u*_*s*_ and a mention candidate *u*_*m*_ pair, we try to predict whether *u*_*m*_ will give a response to the message. This prediction task can be formulated as a binary classification problem. Firstly, based on the observed interdependence between the response probability and the network structure, we extract two types of factors which would affect the prediction performance: (a) Structure factors, including whether or not *u*_*s*_ follows *u*_*m*_ and whether or not *u*_*m*_ follows *u*_*s*_; (b) Influence factors, including the logarithmic of the number of followers of *u*_*s*_ and *u*_*m*_ respectively, the average number of forwardings for each message from *u*_*s*_ and *u*_*m*_ respectively. In addition, according to our previous studies [53], we also consider: (c) Content factors, including whether or not the message contains an embedded URL, whether or not the message is annotated with certain events. Then, we employ three widely used machine learning models for classification task: Support Vector Machine with an RBF kernel (*SVM-RBF*), Linear Regression (*LR*), and Gradient Boosted Decision Trees (*GBDT*).

**Fig 5:**
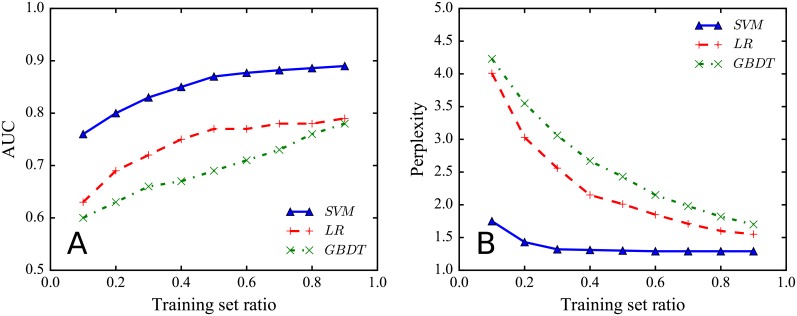
Prediction performance. **(A)** AUC of the three algorithms. AUC measures the area under the ROC curves. **(B)** Perplexity of the three algorithms when predicting response behaviors, against the training set ratio.

To evaluate the prediction performance, we adopt two widely used metrics for classification task: *AUC* and *perplexity*. A higher AUC and a lower perplexity indicate better prediction performance. See Section Materials and methods for details. Fig 5A reports AUC. We find that the *SVM-RBF* classifier obtains the best performance, raising AUC to nearly 90%. We then report perplexity on the testing set with respect to the training set ratio. As shown in Fig 5B, the *SVM-RBF* classifier also achieves the lowest perplexity among all tested classifiers. These results indicate that because of the complexity of the factors that would affect individual’s response behavior, it is more suitable to capture them using a nonlinear feature space mapping. Therefore, we select the *SVM-RBF* classifier to further evaluate the importance of the proposed factors.

Furthermore, in order to analyze how each factor contributes to the prediction, we design a contrast experiment by eliminating one factor at a time and observe how the prediction performance changes. Here we use the *SVM-RBF* classifier for training and testing. We take 50% of all the samples as the training set and the rest 50% as the testing set. As shown in Table 1, we find that when we leave out the content factors (No_Content), the AUC suffers from a 4.6% decline and the perplexity suffers from a 4.2% increase. This finding shows us that the message content can affect a be-mentioned user’s response behavior, but the effect is very limited. One possible explanation is that because the length of each message in micro-blogging network is restricted to no larger than 140 characters, it is still a challenge to reveal the semantics from sparse and noise short texts [57]. In comparison, when we take out the influence factors (No_Influence) from our model, the AUC decreases 9.1% and the perplexity increases 8.5%. This result indicates that the interpersonal influence plays a more important role than message content in information diffusion, which is consistent with empirical findings in previous works [13, 14, 19]. More importantly, when we eliminate the structure factors (No_Structure), we observe a 29.9% decrease of the AUC and a 26.2% increase of the perplexity. This result shows that although message content and social influence help to improve the response prediction result, the structure factors play a much more significant role in the prediction. This result is consistent with our empirical findings about the mention effect of network structure in previous section. The network structure among users, such as structural diversity or structural hole, plays a key role in predicting the individual’s behavior that underlies the social contagion processes [34, 58].

**Table 1:** Comparison on how different factors affect the performance.

	ALL	No_Content	No_Influence	No_Structure
AUC	0.87	0.83	0.79	0.61
Perplexity	1.30	1.35	1.41	1.64

ALL: all factors are combined. No_Content: content factors are eliminated. No_Influence: influence factors are eliminated. No_Structure: structure factors are eliminated. We can observe that although message content and user influence help to improve the response prediction result, structure-dependent factors play a much more significant role in the prediction.

## Discussion

In this paper, the key feature of mention in micro-blogging platform has been investigated comprehensively. We conduct our study on a population-scale dataset from the most popular Chinese micro-blogging network, namely Sina Weibo. We study the statistical characteristics of mention in information diffusion represented as diffusion network. In fact, a significant proportion of these cascades contains mentions in content. We find that users with large number of followers on social network would receive much more mentions than those small-degree users, and meanwhile it brings the problem of mention overload. It will not only interrupt user’s daily use of micro-blogs, but also result in frustration and decrease user’s interest in forwarding. These findings provide us insight and guidance in proposing a new recommendation scheme to maximize the spread of influence.

To further investigate the effect of mention in information diffusion, we examine the response probability from the perspective of mention count and network structure respectively. We observe a saturation at around 5 mentions in a message which means that with each additional mention, a message is more and more likely to receive response from the mentioned users, up to a point. Then we study the response ratio among all mentions in a message and observe a peak at 2 mentions. Beyond the mention effectiveness on the mention count in a message, we further examine the response probability with respect to the network structure between users. We find that the response probability is the highest when a reciprocal followship exists between users. Furthermore, one is more likely to receive a target user’s response if they have similar social status. From the perspective of machine learning, the discovered correspondence provides predictive factors to estimate response probability. To illustrate these findings, we propose the response prediction task and formulate it as a binary classification problem. By adopting features including message content, user influence and topological structure between users, a machine learned prediction function is trained. Extensive evaluation demonstrates the effectiveness of discovered factors.

To understand the variation of response probability for different messages, we also classify messages into different categories according to the content and compare the response probability curves of each category. Due to strict length restriction of a message, many different viewpoints and additional context can be expressed through embedded URL sharing and annotated event keywords, which represent important features of the content of messages [59, 60]. As done in our previous studies [53], we classify messages according to their content, i.e., whether containing embedded URLs or event keywords. The comparison of response probability curves indicate that be-mentioned users are not prone to respond messages containing embedded URLs or events (S2 Fig). This could be explained from the perspective of psychology that people in China are sometimes conservative and protective on online social network while facing social events.

As future work, we will devote to deep investigation on the interdependence between the popularity of a message and the structural characteristics of multiple mentioned users. We will further study whether there are some kinds of significant patterns existing in the information diffusion process. Moreover, it is of great interest to model the individual behaviors from the micro-perspective and uncover the information cascading process with behavioral dynamics.

## Materials and methods

### Data

The data used in this paper are collected from Sina Weibo, which is the most popular micro-blogging platform in China. It includes basic information about messages (time, user ID, message ID etc.), mentions (user IDs appearing in messages), forwarding paths, and whether containing embedded URLs or event keywords. In addition, it also contains a snapshot of the following network of users (based on user IDs). This data is also used in our previous studies [53]. It is now available from the WISE 2012 Challenge (http://www.wise2012.cs.ucy.ac.cy/challenge.html). The results we present are produced using messages that were originally posted between July 1, 2011 and July 31, 2011. We cleaned the data by removing inactive users and unpopular messages. We also removed spam users who abnormally forward a single message for hundreds of times. To alleviate the effect from activity pattern of users, we only consider the messages posted between 10am and 10pm per day, which is the active period in Sina Weibo system. In total, there are 2.6 million messages. And for each message, its forwarding information between July 1, 2011 and August 31, 2011 is also collected. Detailed statistics of the dataset is reported in Table 2.

**Table 2:** Data statistics.

Statistics	Value
Num of users	43,378,576
Num of followships	198,347,101
Num of cascades	2,636,198
Num of cascades with ‘@’	393,772
Num of positive (response) examples	119,845
Num of negative (no response) examples	273,925

### Comparison algorithms and evaluation metrics

We denote with a tuple (*u*_*s*_, *u*_*m*_, *d*) the sample that a user *u*_*s*_ (called the source user) mentions another user *u*_*m*_ in a message *d*. Each time *u*_*m*_ sees the message that he has not forwarded before, we say *δ*_*s*,*m*,*d*_ = 1 if *u*_*m*_ forwarded *d*, forming a positive example indicating *u*_*s*_ successfully activates *u*_*m*_ to give a response to *d*; otherwise *δ*_*s*,*m*,*d*_ = 0 for a negative example if *u*_*m*_ neglects *d*.

To compare the performance of response prediction, three mainstream classification algorithms are implemented to estimate and predict response probabilities on all samples, including Support Vector Machine with an RBF kernel, Linear Regression, and Gradient Boosted Decision Trees. Some other widely used models are not compared because those models require exogenous such as message content or user profiles that are absent in this scenario.

In this paper, we use *AUC* and *perplexity* as evaluation metrics. AUC measures the area under the Receiver Operating Characteristic curve, which represents the probability that a model correctly distinguishes a randomly selected positive sample from a randomly selected negative sample. The perplexity measures how the testing samples surprise a trained model, as shown in Eq (1). A higher AUC and a lower perplexity indicate better prediction performance. The definition of perplexity is as follows:

$$\begin{array}{c} perplexity=e^{-\frac{\sum_{(s,m,d)\in D_{test}}\delta_{s,m,d}\ln\hat{P}(\delta_{s,m,d}=1)+(1-\delta_{s,m,d})\ln(1-\hat{P}(\delta_{s,m,d}=1))}{|D_{test}|}}, \end{array}$$ (1)

where *D*_*test*_ represents the testing set, and $\hat{P}(\delta_{s,m,d}=1)$ is the estimated response probability.

## Supporting information

S1 Fig Response ratio *r*(*k*) versus the average degree of be-mentioned users 〈*d*(*u*_*m*_)〉. We classify all messages into two categories: 〈*d*(*u*_*m*_)〉 ∈ [1, 100), and (〈*d*(*u*_*m*_)〉 ∈ [10000, +∞). We observe a peak in response ratio at 2 mentions and then a slow drop in both categories. Moreover, we find that the response ratio of 〈*d*(*u*_*m*_)〉 ∈ [1, 100) is higher than that of (〈*d*(*u*_*m*_)〉 ∈ [10000, +∞).
(EPS)

S2 Fig The variation of response probability for different kinds of messages. (A) response probability *p*(*k*) versus mention count *k* for messages with and without embedded Events. (B) response probability *p*(*k*) versus mention count *k* for messages with and without embedded URLs.
(EPS)
